# The human DDX52 protein is a nucleic acid helicase and strand annealase that promotes cell migration

**DOI:** 10.1042/BSR20253932

**Published:** 2026-01-09

**Authors:** Ashley J. Parkes, Philipp J. Springer, Edward Bolt

**Affiliations:** 1 School of Life Sciences, University of Nottingham, U.K.

**Keywords:** helicase, translocase, annealing, DExD-Box, cancer

## Abstract

DExD-box (DDX) proteins are essential for RNA metabolism and are targets for treatment of cancers and neurodevelopmental disorders. The biochemical mechanisms of many DDX proteins remain unclear, including human DDX52. DDX52 is essential for cell survival and is an emerging biomarker for the onset of metastatic melanoma. In this work, we identified that human DDX52 is an ATP-dependent translocase with 3’–5’ polarity, which can unwind DNA duplexes and DNA/RNA hybrids. Further, DDX52 is a nucleic acid annealase, an activity that requires an N-terminal intrinsically disordered protein region. DDX52 becomes hyperactive at DNA annealing if DDX52 helicase activity is inactivated by mutagenesis. Using CRISPR-Cas9 genetic editing, we generated U2OS cell lines heterozygous for DDX52 (*DDX52*^+/–^), which exhibit growth defects and impaired cell migration, providing direct support for previous suggestions that DDX52 may promote cancer cell metastasis and C-myc regulation.

## Introduction

DExD-box (DDX) proteins comprise a family of putative RNA helicases featuring a highly conserved helicase core. They consist of two RecA-like domains containing a series of nine conserved motifs associated with ATP hydrolysis and nucleotide binding, most notably their signature Asp-Glu-X-Asp motif adjacent to an ATPase active site (the ‘D-E-x-D box’). The largest family of superfamily II helicases, DDX helicases are widely associated with RNA processing during transcription and translation, including within ribosome biogenesis, RNA export and facilitating local unwinding of short RNA [1]. However, members of the family are also associated with multifunctional roles in innate immunity, genome stability and transcription, reflecting their amino acid sequence diversity beyond the DDX helicase core [2–4]. This sequence diversity is most variable in the N- and C-termini regions of DDX proteins, which are notable for intrinsically disordered regions that are poorly characterised [4]. Loss or malfunction of individual DDX proteins is associated with a range of human cancers and neurodevelopmental disorders [5,6].

The human DDX helicase DDX52 is one such example – overexpression of DDX52 is a prognostic marker associated with poor recovery within human liver and lung cancers [7,8], and knockdowns of DDX52 suppress proliferation within malignant melanoma and prostate cancer cells through regulation of c-myc signalling, identifying DDX52 as a potential therapeutic target [9,10]. Antiviral properties of DDX52 against Myxoma virus have also been reported [11], along with associations of DDX52 for normal cellular development, including spermatogenesis [12,13]. However, the biochemical activity of human DDX52 has not been studied. Predictions for DDX52 function are based on similarity with the homologous yeast protein Rok1, which when depleted causes blocked 18S rRNA synthesis through inhibition of pre-rRNA processing sites A0, A1 and A2 [14,15]. Further, phosphorylation of human DDX52 residues in response to DNA damage is implicated in DNA repair [16,17].

When investigating purified DDX52, we identified (a) both nucleic acid helicase and annealing activities, (b) a necessary DNA binding region within a predicted IDPR of DDX52 and (c) mutations causing hyperactive annealing. Homozygous deletions of the *DDX52* gene were not possible using CRISPR-Cas9 in human U2OS cells, consistent with essentiality of DDX52 suggested in Human Cancer Dependency Mapping [18], but *DDX52* ^–^/*DDX52* ^+^ heterozygotes were inhibited in cellular proliferation and migration, supporting the relevance of DDX52 as a target to control metastasis.

## Results

### Human DDX52 binds to DNA and RNA and is a helicase/translocase with 3’ to 5’ directionality

**Figure 1:**
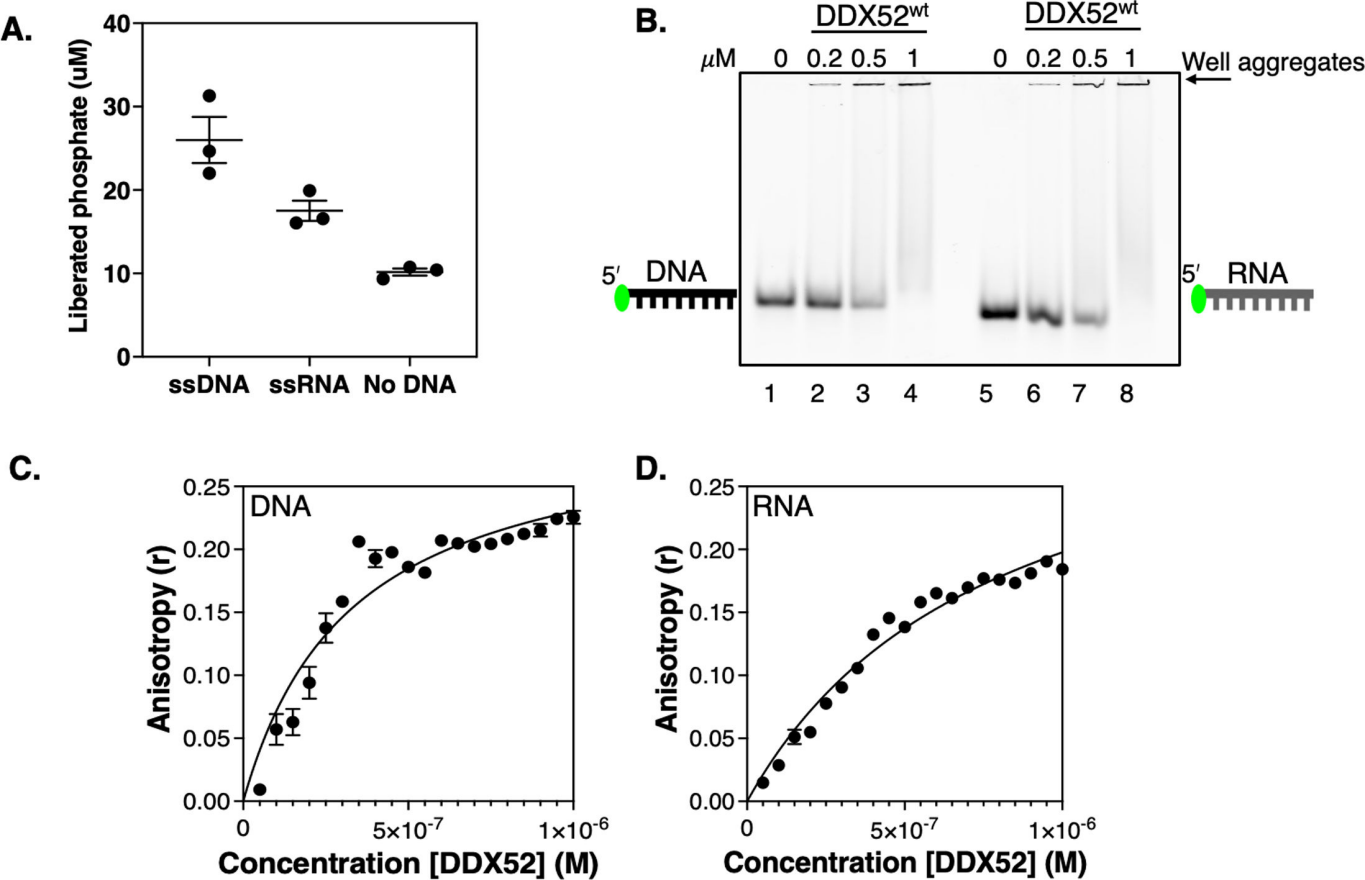
Biochemical properties of human DDX52 protein. (A) Malachite green reporter assay measurements of DDX52 (200 nM) ATPase activities in the presence or absence of single-stranded DNA (ssDNA) and single-stranded RNA (ssRNA) oligonucleotides (25 nM). Data points show plots are from three independent experiments, with bars representing standard error from the mean. (B) EMSAs of DDX52 binding to 5’ end-labelled ssDNA (lanes 1–4) and ssRNA (lanes 5–8) oligonucleotides (25 nM) of equivalent sequence, forming in-well aggregates under these assay conditions. DDX52 protein concentrations are shown on the panel. (C) Anisotropy measurements of DNA as a function of DDX52 concentration (0–1 𝜇M). DNA is a poly-thymine 5′-fluorescein-labelled ssDNA oligonucleotide (40 nM) – see Supplementary Data for full sequences. Data points are means from two independent assays, with error bars showing standard deviation. (D) Anisotropy measurements of RNA as a function of DDX52 concentration (0–1 M). RNA is a poly-uracil 5′-fluorescein-labelled ssRNA oligonucleotide (40 nM). Data points are means from two independent assays, with error bars showing standard deviation.

We purified human DDX52 protein (69 kDa, Supplementary Figure S1A) to assess its interaction with DNA and RNA *in vitro*. ATPase activity of DDX52 was stimulated by 2.6- and 1.7-fold when adding, respectively, a 45-nucleotide single-stranded DNA (ssDNA) or single-stranded RNA (ssRNA) molecule of equivalent sequence, compared with the absence of nucleic acid (Figure 1A). EMSAs of DDX52 binding to the same Cy5 end-labelled DNA or RNA (Figure 1B) did not show a clearly defined DDX52–nucleic acid complex, which would be indicative of stable and discrete binding – instead, protein-dependent DNA and RNA high-molecular-mass aggregates were produced in gel wells, which is inconclusive in this assay as *bona fide* protein–nucleic acid complex. We were able to measure in-solution and in real time the changes in anisotropy of FAM end-labelled DNA and RNA on adding DDX52, which showed moderately higher affinity binding of DDX52 to DNA (*Kd* 0.33 μM) than RNA (*Kd* 0.74 μM) (Figure 1C and D).

**Figure 2:**
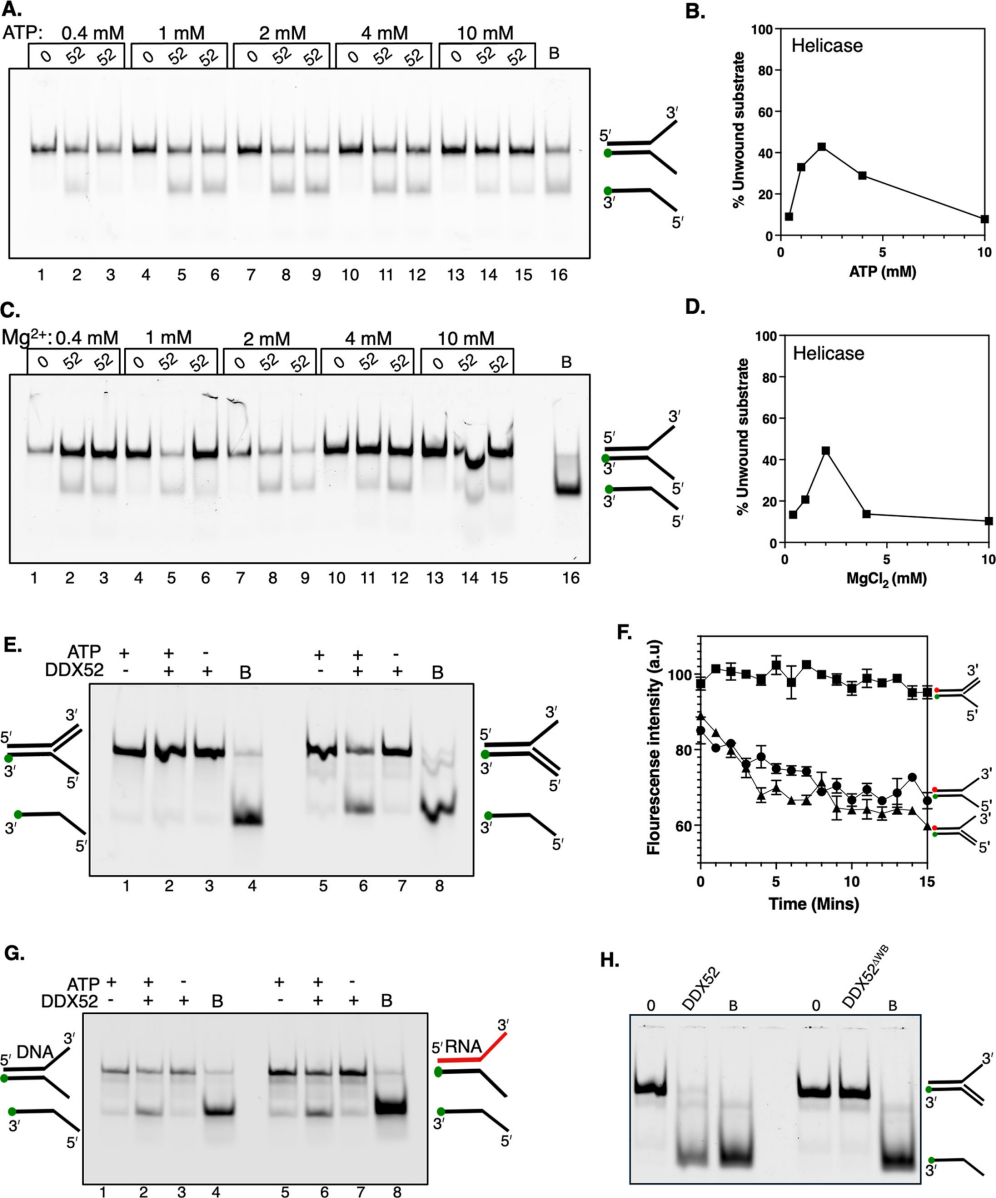
DNA and RNA helicase activity from human DDX52. (A and B) Helicase products from DDX52 protein (200 nM) and a corresponding graph quantifying them as a % of total DNA in reactions. Reactions contained 2 mM of magnesium chloride and variable ATP concentrations, unwinding 3ʹ Cy5-labelled flayed duplex DNA substrate (25 nM), as indicated. Each DDX52 reaction at the given ATP concentration is shown in duplicate, from two independent assays, giving the accompanying graphed data of the mean helicase activity. (C and D) As for panels A and B, except that these reactions titrated ATP from 0.4 to 10 mM as indicated, with magnesium chloride at 2 mM. (E) DDX52 helicase products from ATP-dependent unwinding of 3ʹ Cy5-labelled DNA substrates (25 nM) with either a 3ʹ or 5ʹ single-stranded DNA (ssDNA) flap, as indicated, in reactions containing 2 mM Mg^2+^ and 2 mM ATP. Lane marked B is boiled to show full dissociation of DNA. (F) Fluorescence resonance energy transfer (FRET) measurements of DDX52 (500 nM) unwinding the DNA molecules shown (each 50 nM) featuring 3ʹ Cy5 and 5ʹ Cy3 labels and 5ʹ, 3ʹ and both 5ʹ and 3ʹ flaps as indicated. The data are mean values with standard error. (G) Helicase products indicating ATP-dependent unwinding by DDX52 (500 nM) of 3ʹ Cy5-labelled substrates (25 nM), a DNA duplex (lanes 1–4) and a comparable DNA/RNA hybrid (lanes 5–8). Lane B is a boiled reaction. (H) Helicase products indicating ATP-hydrolysis dependent unwinding of 3ʹ Cy5-labelled DNA (50 nM) by wildtype DDX52 (DDX52^WT^, 500 nM) compared with the Walker B inactivated mutant, DDX52^ΔWB^ (500 nM). Lane marked B is a boiled reaction.

As a DDX family helicase, as predicted from amino acid sequence similarities, we tested if DDX52 has nucleic acid translocase/helicase activity *in vitro*. We began by using as a potential substrate a flayed duplex DNA, which provides both ssDNA and double-stranded DNA (dsDNA) regions, and with accessible 3’ and 5’ ends of both ssDNA and duplex DNA (Figure 2A). This simple substrate therefore provides multiple potential sites for DDX52 loading. Firstly, we tested for helicase activity of DDX52 (200 nM) in duplicate reactions comprising 2 mM magnesium chloride, with ATP titrated from 0.4 to 10 mM (Figure 2A and B). This showed 2 mM ATP to be optimal for DDX52 to dissociate the flayed duplex into ssDNA. Repeating these assays by varying magnesium chloride from 0.4 to 10 mM, in 2 mM of ATP, confirmed 2 mM magnesium chloride as optimal for DDX52 helicase activity (Figure 2C and D). We therefore used 2:2 mM ATP:magnesium chloride for subsequent analysis of helicase activities.

Next, by adding a third DNA strand to the flayed duplex to form partial forks (Figure 2E), we observed that dissociation of DNA by DDX52 was ATP-dependent as expected, but that DNA unwinding required that a 3’ ended single-stranded region − the equivalent ‘mirror-image’ fork with a 5’ ended single-stranded region was not unwound at all (Figure 2E, compare lanes 2 and 6). This specificity for DDX52 unwinding from a 3’ ended fork was made clear in solution in real time using fluorescence resonance energy transfer (FRET) − FRET between base-paired DNA strands decreases as DNA strands in substrates are dissociated, apparent for only the flayed duplex and fork with the 3’ ssDNA loading site, with no dissociation of the 5’ ssDNA ended fork (Figure 2F).

Having identified substrate preference for ATP-dependent unwinding by DDX52, we substituted the 3’ ended DNA strand of the flayed duplex for equivalent RNA and observed ATP-dependent unwinding in agreement with similar DNA- and RNA-stimulated ATPase activities (Figure 2G, compare lanes 2 and 6). Finally, no helicase activity was detected against the 3’ ended fork when replacing DDX52 with DDX52Δ^WB^ − a mutant lacking a functioning ATPase motif that is unable to hydrolyse ATP − confirming ATP-dependent helicase activity (Figure 2H). We conclude that purified DDX52 protein has directional 3’ to 5’ ATP-dependent DNA and RNA translocation activity, as a helicase.

### Dissection of DDX52 protein reveals DNA binding and annealing regions

**Figure 3:**
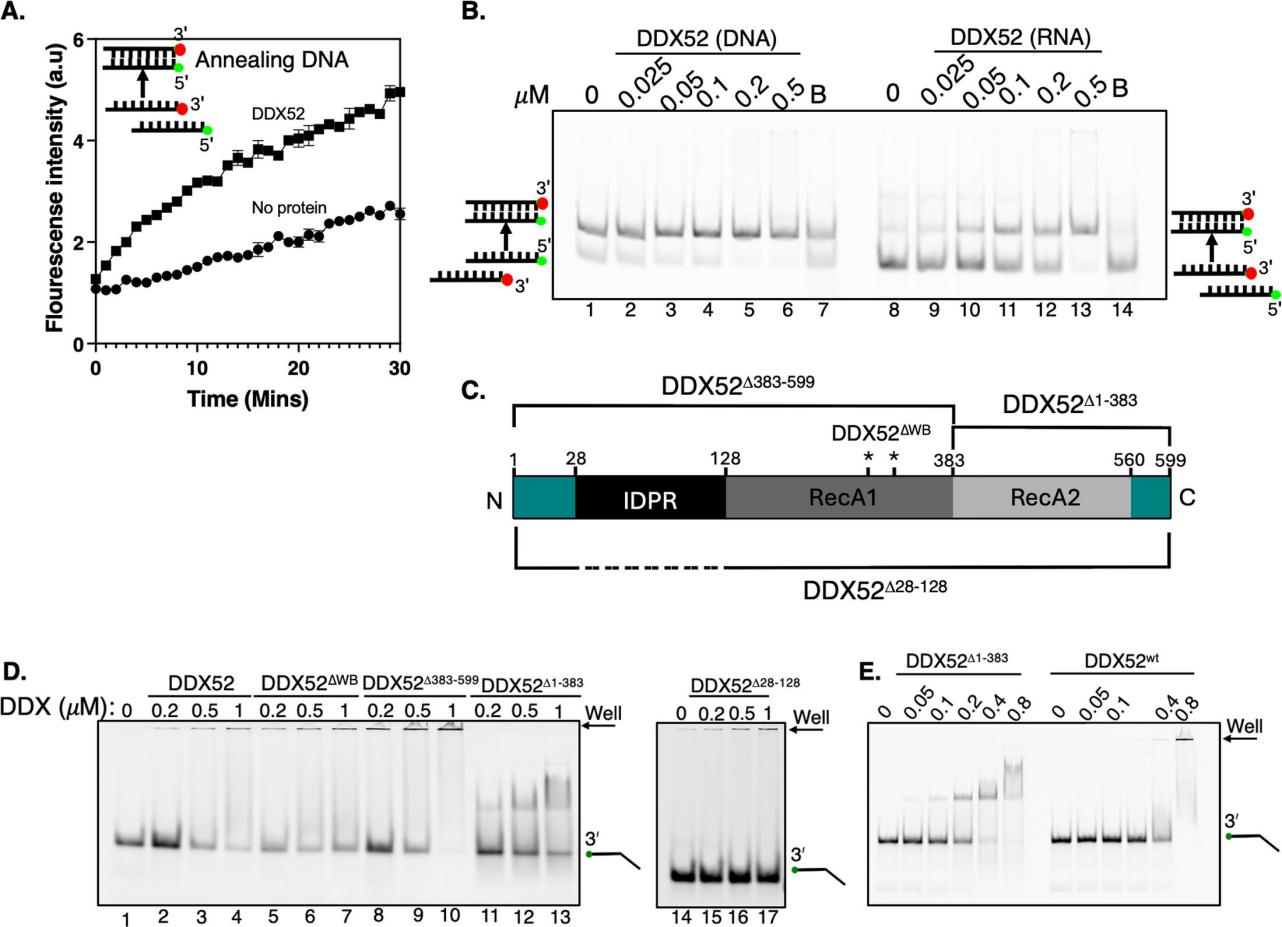
Nucleic acid annealing by DDX52. **(A)** Fluorescence resonance energy transfer (FRET) measurements showing increases in fluorescent intensity on incubation of two single-stranded DNA (ssDNA) molecules (50 nM) labelled with 3ʹ Cy5 and 5ʹ Cy3, in the presence of DDX52 (500 nM) and without DDX52 as indicated. (B) Gel to visualise annealing products from complementary DNA (lanes 1–7) or RNA (lanes 8–14) oligonucleotides (20 nM each) formed in reactions containing DDX52 at concentrations indicated. (C) Schematic of human DDX52 protein to illustrate the mutants studied in this work. The RecA1 and RecA2 domains, the intrinsically disordered protein region (IDPR) and the sites of the Walker B mutations (D318A and D321A, DDX52Δ^WB^) are shown with asterisks. Also indicated, with lines above and below the cartoon, are the deletion mutant proteins that are described in the main text. (D) EMSAs of binding DDX52 wildtype and DDX52 mutant proteins as indicated (0, 200, 500 nM and 1 μM) to a 5ʹ Cy5-labelled ssDNA oligonucleotide. (E) EMSA comparing the significantly altered binding to DNA (25 nM) of one DDX52 deletion mutant when compared with wildtype DDX52, at protein concentrations as indicated above the panel.

The DDX52 protein homologue in yeast, Rok1, forms RNA duplexes from single strands of RNA [19]; therefore, we tested if DDX52 can do this, using complementary RNA and DNA strands end labelled with either Cy5 or Cy3 to detect annealing through FRET, in solution and in real time. DDX52 was indeed proficient at annealing DNA strands in the absence of ATP-Mg^2+^, observed as increased FRET from base-pairing DNA (Figure 3A), with both DNA:DNA and DNA:RNA annealing also visible when reaction products were electrophoresed in-gels (Figure 3B).

We then tried to determine regions of DDX52 required for helicase and annealing activities. Guided by structure and IDPR predictions for DDX52 (Supplementary Figure S2), we generated and purified mutant proteins lacking parts of the DDX52 protein structure (Figure 3C and Supplementary Figure S3). These DDX52 proteins lacked either the RecA2 C-terminal amino acids 383–599 (44.5 kDa, called DDX52Δ^383–599^) or the RecA1 domain and the major predicted IDPR (24.5 kDa, called DDX52Δ^1–383^), or only the predicted IDPR region (residues 28–128, 57.5 kDa, called DDX52Δ^28–128^). In EMSAs, the DDX52Δ^383–599^ and DDX52^WB^ proteins bound to DNA similarly to wildtype DDX52, as predominantly in-well protein–DNA aggregates (Figure 3D, lanes 1–10). However, DDX52Δ^1–383^ showed unusually robust protein–DNA complex with no in-well aggregation (Figure 3D, lanes 11–13). This indicated that the N-terminal RecA1-IDPR region of DDX52 is required for DDX52 protein–nucleic acid aggregation. Further, DDX52Δ^28–128^ showed very weak DNA binding in the EMSAs compared with wildtype DDX52 (e.g. Figure 3D, compare lanes 3 and 4 with 16 and 17, representing 0.5 and 1.0 μM of each protein). This indicates that the IDPR region is required for proficient DNA binding by DDX52. The contrasting DNA binding by DDX52Δ^1–383^ and wildtype DDX52 was readily observed in EMSAs, utilising a broad range of protein concentrations (Figure 3E).

**Figure 4:**
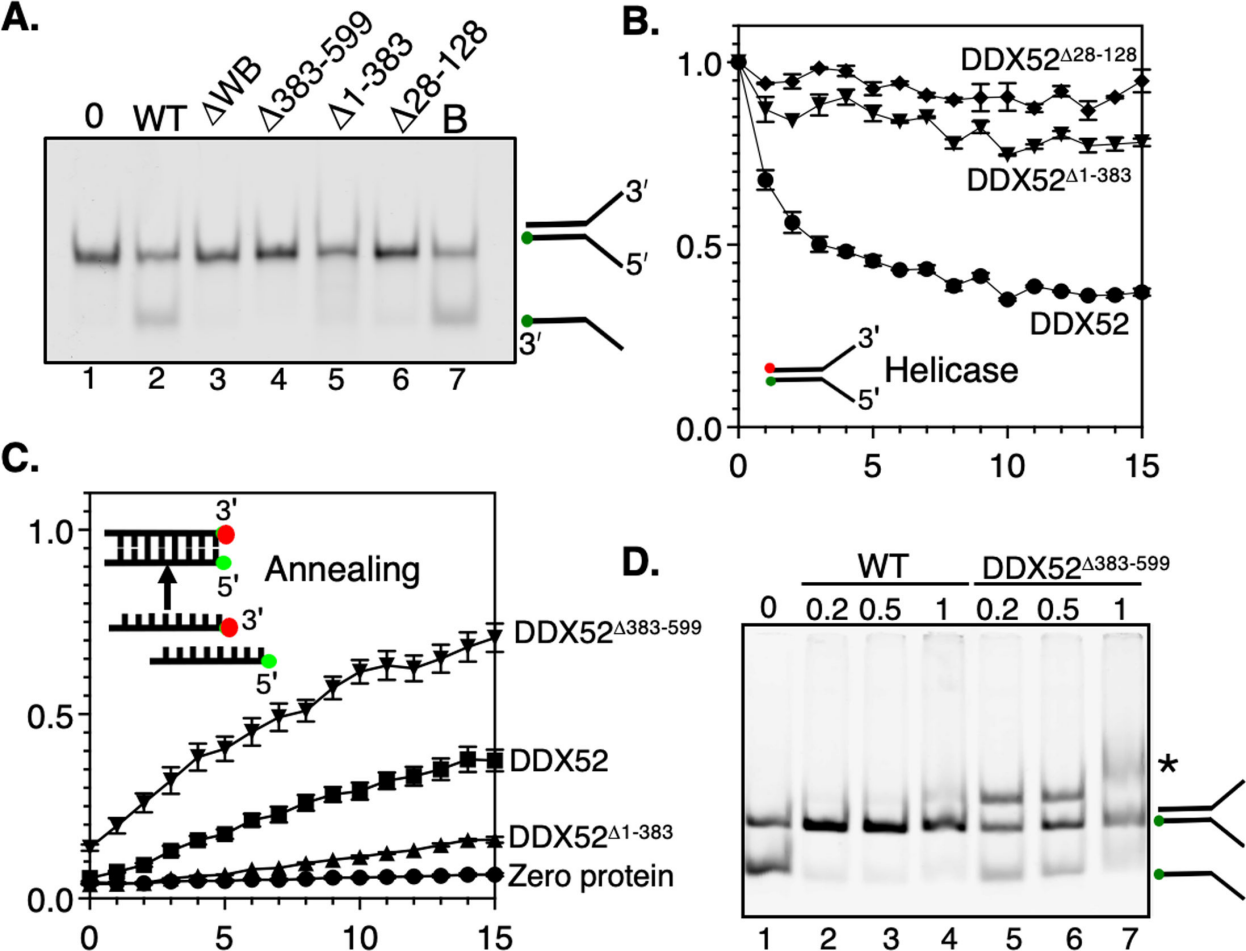
Characterisation of DDX52 domain ‘dissection’ mutants. **(A)** Gel showing helicase products from wildtype DDX52 and mutants (200 nM) on a Cy5-labelled flayed duplex in reactions containing 2 mM Mg^2+^ and 2 mM ATP. Lane marked B is for a boiled reaction. (B) Fluorescence resonance energy transfer (FRET) measurements of wildtype DDX52 (500 nM) helicase activity, compared with the Δ1–383 and Δ28–128 (500 nM) mutant DDX52 proteins. Data points show mean values from two independent reactions, with bars representing standard error. (C) FRET measurements of wildtype DDX52 (800 nM) DNA annealing activity, compared with the Δ1–383 and Δ383–599 (800 nM) mutant proteins. Data points show mean values from two independent reactions, with bars representing standard error. (D) Gel comparing DNA annealing products formed by wildtype DDX52 (lanes 2–4) and DDX52Δ^383–599^ (lanes 5–7), from two complementary strands of Cy5-labelled DNA. The star highlights the formation of additional annealing product by the mutant DDX52 protein.

The DDX proteins lacking one of their two RecA domains (DDX52Δ^1–383^ and DDX52Δ^383–599^) or the DDX52^WB^ protein were, as expected, inactive as helicases because they lack critical ATPase motifs, summarised using end-point assays and real-time FRET in Figure 4A and B. DDX52Δ^28–128^ was also inactive as a helicase, consistent with much-reduced DNA binding activity (Figure 4A and B). However, the DDX52Δ^383–599^ protein showed hyperactive DNA annealing activity compared with wildtype DDX52, measured in real time by FRET over a range of protein concentrations (Figure 4C and Supplementary Figure S4). This reveals that DNA binding modules within the IDPR and RecA1 regions of DDX52 (amino acids 1–383) control annealing reactions, counterbalanced by the RecA2 domain, which is absent from DDX52Δ^383–599^. Consistent with this, and its much-reduced DNA binding, DDX52Δ^28–128^ showed much reduced DNA annealing activity.

Interestingly, when we visualised on gels the annealing products of hyperactive annealing from DDX52Δ^383–599^, we saw formation of an additional higher-molecular-mass DNA band, which may indicate triplex DNA (Figure 4D). This DNA was clearly abundant at as little as 200 nM of DDX52Δ^383–599^, whereas it is only weakly observed from wildtype DDX52 at 1 μM (e.g. compare lanes 4 and 5). We conclude that the predicted IDPR residues of DDX52 are required for DNA binding – discussed more below – and that networks of DDX52 proteins bring DNA into close proximity to promote annealing of complementary DNA.

### Human DDX52 promotes cell migration

**Figure 5:**
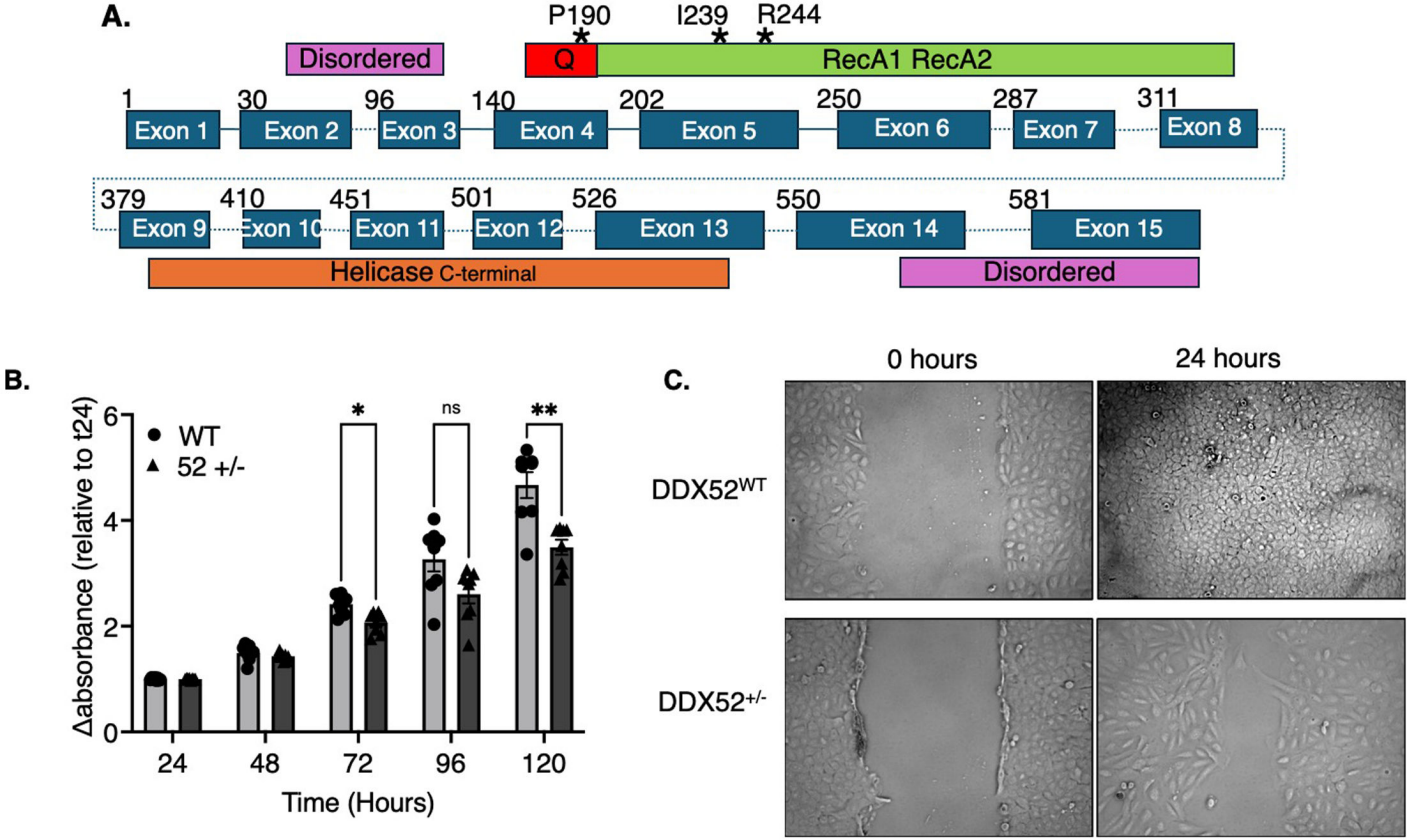
Cell proliferation of wildtype (WT) and DDX52*^+/–^* U2OS cells. **(A)** Schematic showing the organisation of *DDX52* exons matched with the corresponding regions of the DDX52 protein: The predicted disordered N-terminal region spanning exons 2 and 3, the DDX-helicase core regions (exons 4–13) and the C-terminal predicted disordered region (exons 14 and 15). Marked are the sites of codons encoding the amino acids Pro-190, Iso-239 and Arg-244, which were central to each of the three guide RNAs used to target *DDX52* in cells, as described in Methods and Supplementary Table S5. Targetting of Iso-239 generated deletion that was viable to create the *DDX52* ^–/+^ cells. (B) WST-1 cell proliferation measurements (A_440 nm_) comparing *DDX52* ^+/−^ with *DDX52* ^+/+^ U2OS cells. The graph shows means with standard deviation error bars from eight repeats. Statistical analysis was performed using two-way ANOVA followed by Sidak’s multiple comparison test. *P<0.05, ***P<0.001 (compared with WT group). (C) Images of a representative wound-scratch healing assay comparing the rate of migration of *DDX52* ^+/−^ and *DDX52* ^+/+^ U2OS cells from 0 to 24 hours. Images were captured using phase-contrast microscopy at ×10 magnification.

The DepMap database (https://depmap.org/portal/ and https://tubic.org/deg/public/index.php) of CRISPR-Cas9 and RNAi depletion studies in human cells indicate essentiality of human DDX52 [18,20]. Consistent with this, when we targetted *DDX52* exons 4 and 5 with guide RNA (gRNA) for CRISPR-Cas9 knockout in human osteosarcoma (U2OS) cells (Figure 5A), the only viable cells were heterozygous (*DDX52* ^+/–^), containing a single base-pair deletion – see the Methods for a full description of how this cell line was generated and Supplementary Data (Supplementary Figure S5) for their validation. The top three predicted off-target sites from analysis by CRISPOR [21] were sequenced in this cell line, and no off-target mutations were detected at those sites. The *DDX52* ^+/–^ U2OS cells were used to measure cell proliferation and migration compared with *DDX52* ^+/+^ cells. Cell viability as measured by WST-1 showed growth of *DDX52* ^+/–^ U2OS cells to be significantly reduced compared with wildtype cells, especially after 120 hours of growth (Figure 5B). This was supported using scratch-wound healing assays, showing that the actual migration of *DDX52* ^+/–^ cells was at a slower rate than *DDX52* ^+/+^ cells when observed over 72 hours – wildtype cells recovered within 24–48 hours, whereas *DDX52* ^+/–^ cells fully recovered between 48 and 72 hours (Figure 5C). This supports the vital role of DDX52 in cell viability and migration, highlighting its potential as a target in preventing tumour metastasis and supporting studies where DDX52 knockdowns inhibited proliferation of the highly metastatic melanoma.

## Discussion

DDX52 is thought to be an ATP-dependent helicase, based on its amino acid sequence homology to the characterised yeast helicase, Rok1 [19]. In cells, it has a likely role in ribosome biogenesis and is associated with tumourigenesis by controlling expression of the oncogene c-myc [9,10]. However, there is little information about the biochemical properties of DDX52. Our data show that DDX52 is proficient as a DNA and RNA helicase, and that it has DNA and RNA strand annealing activity − Rok1 is also an annealing enzyme [19]. By generating DDX52 proteins lacking various regions, we were able to begin to investigate how the protein controls annealing and helicase functions. Generating these mutant proteins was challenging, evidenced by varying yields of the final purified proteins (Supplementary Figure S3), and we acknowledge that generating them using *Escherichia coli* precludes any influence of potential post-translational modifications to DDX52 in human cells. Nonetheless, the robustly hyperactivated annealing activity of DDX52Δ^383–599^, which lacks the RecA2 domain, was accompanied by DNA binding that was similar to the DDX52 wildtype protein, providing confidence in the quality of purified DDX52Δ^383–599^. These data indicated that the N-terminal region of DDX52 controls annealing, antagonised by the C-terminal RecA2 domain, an insight supported by the observation that deleting the N-terminal IDPR (DDX52Δ^28–128^) abolished DNA binding. Therefore, it suggests that the function of the N-terminal IDPR of DDX52 is to draw together DNA strands for annealing. This idea may be supported by an unusually high frequency of arginine and lysine residues (20% of total residues) within this IDPR. How antagonistic annealing/helicase functions may be controlled in cells is not clear from our data, but it is probably determined by interactions with other proteins or post-translational modifications. RNA annealing by Rok1, the *Saccharomyces cerevisiae* homologue of DDX52, is stimulated by interaction with Rrp5 protein [19] – Rrp5 is also present in humans, which is therefore likely to have the same effect on DDX52.

Perhaps significantly, hyperactive annealing by DDX52Δ^383–599^ also readily formed a higher-order DNA structure resembling triple-helical (H-DNA) DNA; the formation of triplex DNA within promoter elements has been linked with promoting the transcription of c-myc [22,23]. This may point to conditions under which wildtype DDX52 in cells can therefore stimulate c-myc expression. Our findings that heterozygosity of DDX52 slowed cell proliferation and migration within tumour cell lines (Figure 4) support previous associations with cancer [7,9] and highlight it as a biomarker and therapeutic target within cancer. We hypothesise that the activity of DDX52 as an annealase is functionally linked with transcriptional regulation of c-myc through promoting the formation of H-DNA, with helicase and annealase activities of DDX52 regulated according to ratios of ATP and ADP in the local environment, similar to that seen in the DDX DED1 [24]. The present paper highlights DDX52 as a viable therapeutic target in cancer, with increased formation of H-DNA within DDX52 variants potentially positing DDX52 as a cancer driver gene.

## Methods

### Human DDX52 proteins

A list of plasmids used within the present paper can be found in Supplementary Table S1. The 1800-nucleotide ORF encoding human DDX52 (CCDS: CCDS11323.1) was gene-optimised and synthesised for expression in *E. coli* by GeneArt (Life Technologies) prior to insertion into pETDuet-1 through PCR cloning into BamHI and HinDIII restriction sites. DDX52 mutants were created using DDX52 expression vector using primers listed in Supplementary Table S2. Purification steps were at 4°C, unless otherwise stated. Biomass was recovered by centrifugation (4500×g, 10 minutes), resuspended in buffer A (25 mM Tris-HCl pH 7.5, 500 mM NaCl, 25 mM imidazole, 10% glycerol) and supplemented with 1 mM PMSF. Cells were lysed by sonication, centrifuged (35,000×*g*, 35 minutes) and supernatant was loaded onto a 5 mL HisTrap™ HP column (GE Healthcare) at ambient temperature prior to elution in a gradient of 25–400 mM imidazole. DDX52 fractions were confirmed through SDS-PAGE, pooled and dialysed into pre-chilled buffer B (25 mM Tris-HCl pH 7.5, 200 mM NaCl, 10% glycerol) for 16 hours. Dialysate was loaded onto a HiTrap Heparin HP column (1 mL) for further purification over a 0.2–1.0 M NaCl gradient. DDX52-positive fractions were identified, pooled and dialysed as previously described into buffer C (25 mM Tris-HCl pH 7.5, 200 mM NaCl, 35% glycerol). DDX52 and mutants were aliquoted and snap-frozen using dry ice prior to storage at –80°C. Protein concentrations were determined from UV absorption readings (280 nm) in a DeNovix NanoDrop spectrophotometer using Beer–Lambert’s law and extinction coefficients as determined from ProtParam [25].

### Assays and substrates: ATPase, EMSAs and activity assays

DNA and RNA strands used within assays were custom-synthesised (Sigma-Aldrich) and are listed in Supplementary Table S3. ATPase activity was measured using BIOMOL Green (Enzo Life Sciences) malachite green colourimetric detection kit in accordance with the manufacturer’s instructions. FRET measurements for helicase assays used Cy5 and Cy3 end-labelled DNA to form a FRET pair to observe decreasing energy transfer during substrate unwinding. This was measured as changes in sample emission at 590 nm at 37°C in a FLUOstar Omega microplate reader (BMG Labtech) at 1-minute intervals for 30 minutes. Gains were adjusted to the highest signal (Cy3 control) prior to the addition of protein. Fluorescent polarisation methods incubated protein with 40 nM of FAM-labelled poly(T) and poly(U) ssDNA and ssRNA substrates. Data analysis was performed in GraphPad Prism software that used the single-binding mode to calculate anisotropy values (*R*-values) for DNA and RNA from the following equation:


$$R\text{=}\frac{Ia\text{+}Ib}{Ia\text{+}2Ib}$$

Gel-based assays used Cy5 end-labelled DNA or RNA (as referenced in figures) imaged using an Amersham Typhoon 5 biomolecular imager (laser LD635, filter-set Cy5 Fltr 670BP30). EMSAs incubated DDX52 proteins with substrate (25 nM) in buffer HB (20 mM Tris-HCl pH 7.5, 7% glycerol, 100 ug/ml bovine serum albumin) supplemented with 25 mM DTT for 30 minutes at 37°C prior to direct loading on a 5% TBE acrylamide gel for electrophoresis in a Protean II tank (Bio-Rad) for 90 minutes migration. Gel-based activity assays were carried out using 0.5 M respective DDX52 protein in buffer HB supplemented with 2 mM ATP, 2 mM magnesium chloride and 10 mM DTT, unless stated otherwise, incubating DDX52 proteins with Cy5 end-labelled nucleic acids (25 nM) for 30 minutes at 37°C prior to halting the reaction through the addition of proteinase K as a 2 mg/mL solution in 2.5% SDS and 200 mM EDTA. Reactions were visualised on 10% native TBE gel following 60 minutes migration in 1× TBE buffer, or in the case of denaturing gels, a 15% acrylamide TBE gel containing 7 M urea and migration at 10 W for 180 minutes in formamide dye (15% w/v formamide; 4 mM EDTA; 4% v/v glycerol).

### Human cell lines and cell culture

U2OS and derived cell lines used within the present study were routinely cultured at 37°C under 5% CO_2_ in Dulbecco’s modified Eagle medium supplemented with 10% foetal bovine serum, 2 mM L-glutamine and 1% PenStrep (100 U/mL penicillin and 100 μg/mL streptomycin), hereinafter referred to as complete medium. All experiments were carried out at low passage.

### CRISPR-Cas9 genetic editing of *DDX52*

The Alt-R™ CRISPR-Cas9 workflow from Integrated DNA Technologies (IDT) was used to target exons 4 and 5 in *DDX52* of human U2OS-derived cells. gRNAs for targetting of *DDX52* were chosen through predictions using the IDT RNA design checker tool and after initial trials, and can be seen in Supplementary Table S5.

Cells were cultured in complete medium to 80% confluency, treated with trypsin, and following a cell count in triplicate were seeded 24 hours prior to transfection at a density of 40,000 cells per well in 24-well plates. Annealing of crRNA and tracrRNA to form gRNA followed the manufacturer’s protocol and was mixed with recombinant Alt-R HiFi Cas9 nuclease in Cas9Plus reagent and serum-free media to form a ribonucleoprotein targetting complex (RNP, 240 nM). Cells were transfected using Lipofectamine™ CRISPRMAX™ (Thermo Fisher Scientific) containing gRNA-Cas9 mix to a final concentration of 11 nM RNP *per* well. An enhanced green fluorescent protein expression vector was used as a transfection control. Cells were incubated for 48 hours prior to detachment and seeding into two 96-well plates at a density of 0.5 cells *per* well. Plates were incubated undisturbed at 37°C under 5% CO_2_ for one week and single colony-forming units identified using an Axiovert S100 microscope and expanded. Genomic DNA was extracted using the PureLink™ Genomic DNA Mini Kit according to the manufacturer’s protocol and quantified using a DeNovix Spectrophotometer: dsDNA concentration = 50 μg/mL × OD260 × dilution factor. For on-target and off-target testing, genomic DNA (100 ng) was PCR-amplified using primer sequences flanking the editing sites, designed by the CRISPOR tool, and listed in Supplementary Table S6, and amplicons were sent for Sanger sequencing; edited amplicons were compared with wildtype amplicons using TIDE analysis suite (http://shinyapps.datacurators.nl/tide/) with parameters set to identify Indels with a maximum size of 50 bp and a *P*-value threshold of 0.001. Successfully edited cells were transferred to vapour-phase liquid nitrogen for long-term storage.

### U2OS cell proliferation and migration assays

Cell proliferation was assessed using WST-1 cell proliferation reagent (Sigma-Aldrich) at 24-hour increments over 120 hours. In summary, 4000 cells per well were seeded into 96-well plates and incubated for 24 hours at 37°C under 5% CO_2_. WST-1 reagent (10 µL) was added at each time-point and plates incubated for 4 more hours. Absorbance was recorded at 440 nM using a FLUOstar Omega microplate reader. Background absorbance was corrected using wells containing media-only and cell growth rate assessed relative to Δ absorbance. For migration assays, cells were cultured in 100 mm culture dishes until 90–100% confluency. A scratch in the monolayer was introduced via sterile micropipette tip, cells washed with PBS and imaged (Axiovert S100 microscope, Zeiss). Cells were routinely incubated for 24 hours prior to further washing and imaging.

## Supplementary material

online supplementary material 1.

## Data Availability

Relevant data files have been deposited for public access by copying this URL into a web browser: https://drive.google.com/drive/folders/1Ig4wuDRrkkUv6hVVHbZKPLdP6v9r8Epy?usp=share_link
