# Supplementary material for: The human DDX52 protein is a nucleic acid helicase and strand annealase that promotes cell migration
Source: Biosci Rep. 2026 Jan 9;46(1):BSR20253932. doi: 10.1042/BSR20253932 (PMC12863030; doi:10.1042/BSR20253932)
Supplement: online supplementary material 1. [file bsr-46-1-BSR20253932-s001.docx]

**Supplementary Figures**

**Figure S1:**

Purified DDX52 protein (5 mg) after Coomassie-blue straining, with size marker ladder in kilodaltons.

**
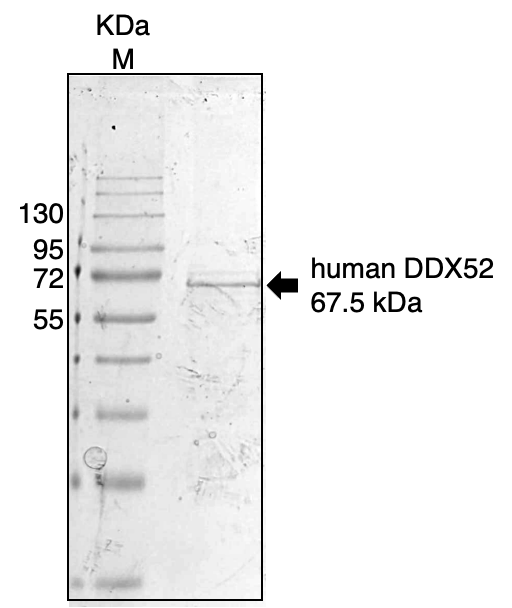
**

**Figure S2:**

**A:** Alphafold predictions of protein folding in human DDX52 (grey) with DExD-box motifs highlighted according to the colour scheme indicated in supplementary item 1B. N (M1) and C-terminal (S599) residues are indicated for reference.

**B:** Summary of the conserved DExD-box domains and their constituent amino acids in DDX52. Colours are depicted as portrayed in the Alphafold predicted structure in supplementary item 1A.

**C:** Predictive plot from fIDPnn database indicating IDPR and predicted protein, DNA and RNA binding regions.

**
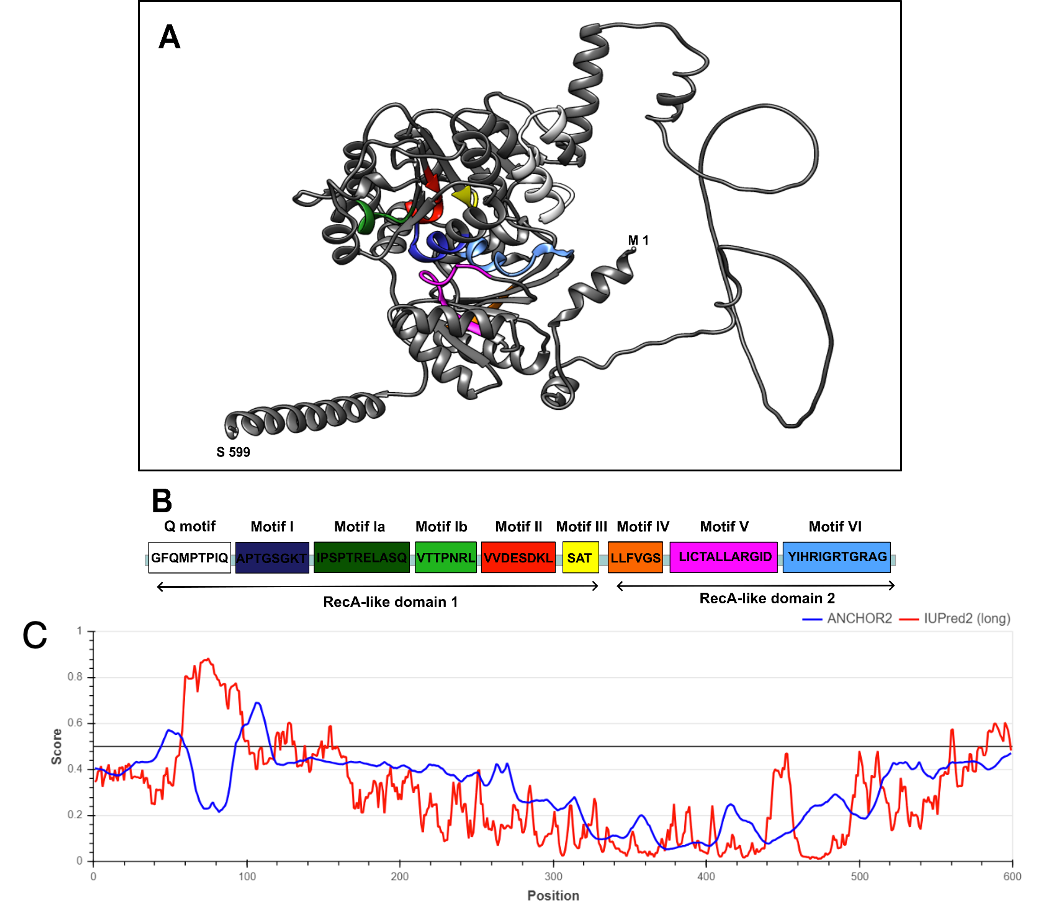
**

**Figure S3:** Coomassie-blue stained gel showing purified wild type and mutant DDX52 proteins (5 μg each) used within this study, with size marker ladder in kilodaltons.


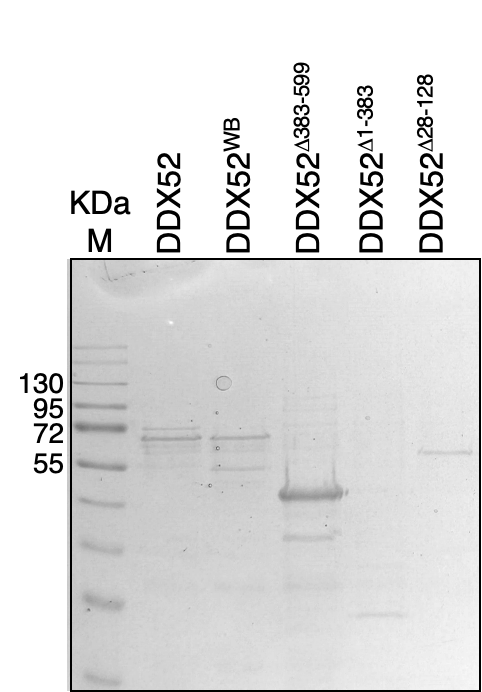


**Figure S4:**

FRET measurements of DNA annealing activity of DDX52 WT compared with the Δ1-383 and Δ383-599 mutant proteins. Concentrations were (nM) 800(◆) 400(▼) 200 (▲) 100 (▪️) and 0 (⬤). Data points show mean values of two independent experiments with bars representing standard error.

**
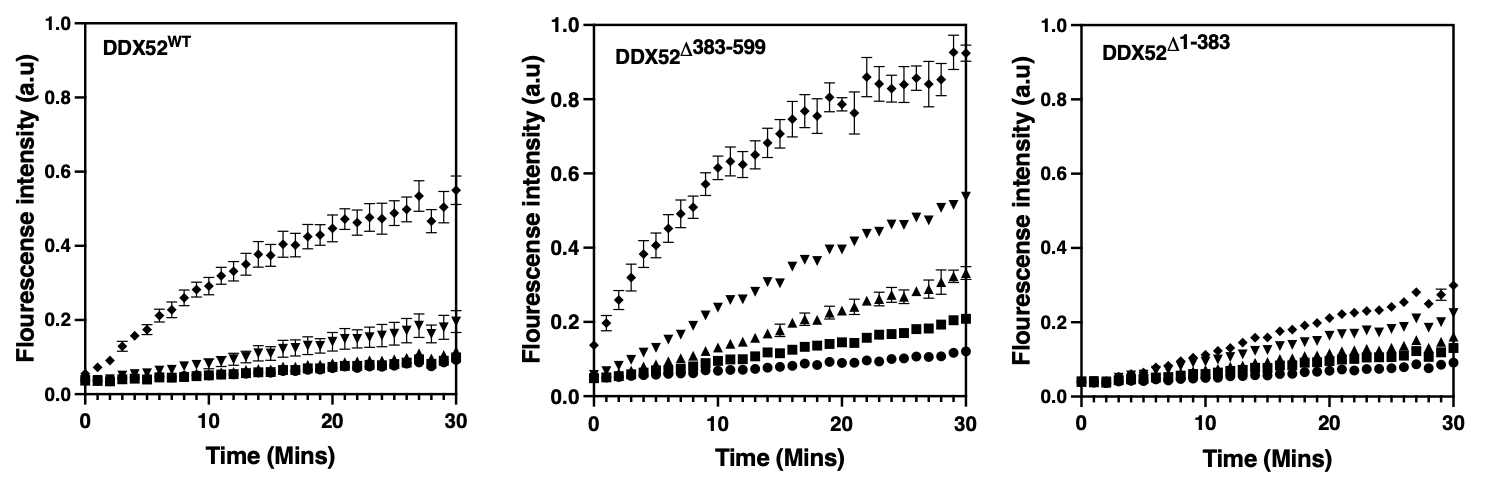
**

**Figure S5:**

**
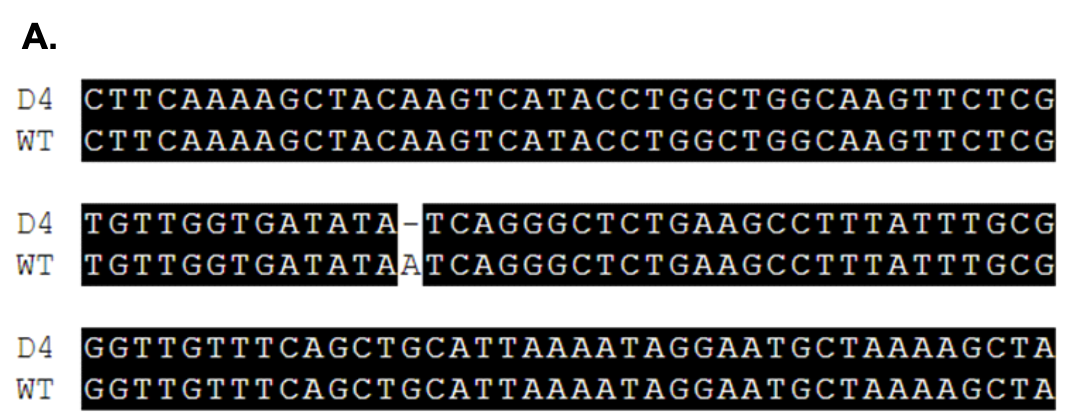
**

**
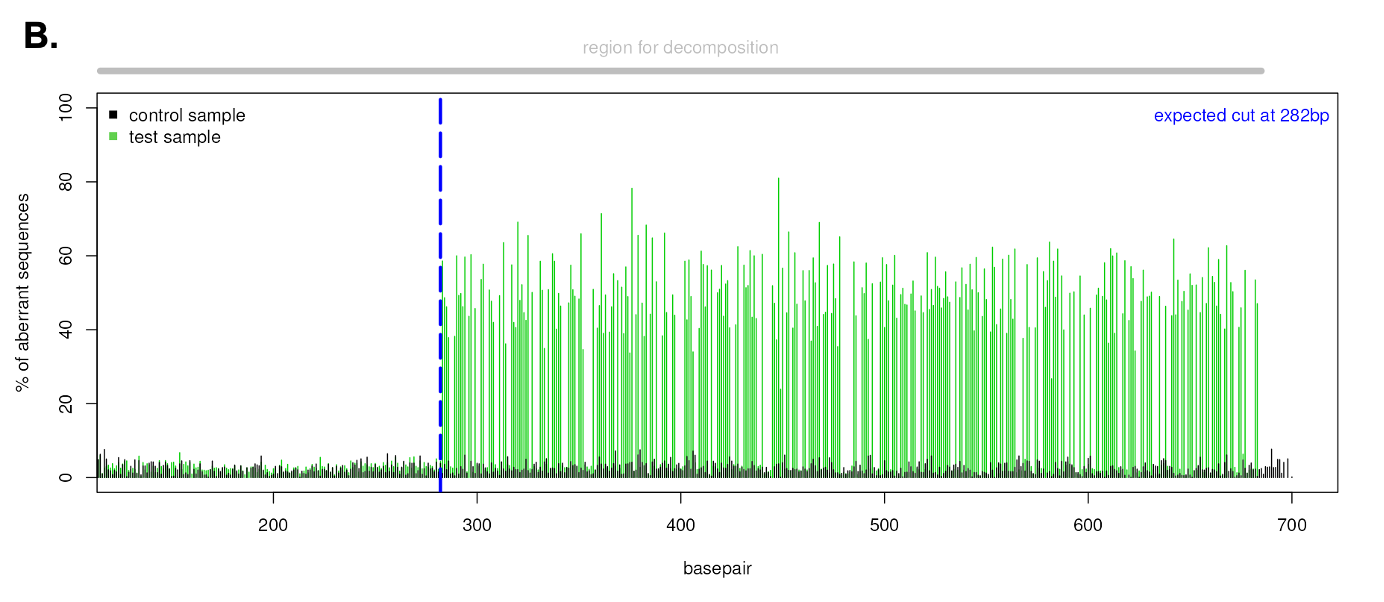
**

**
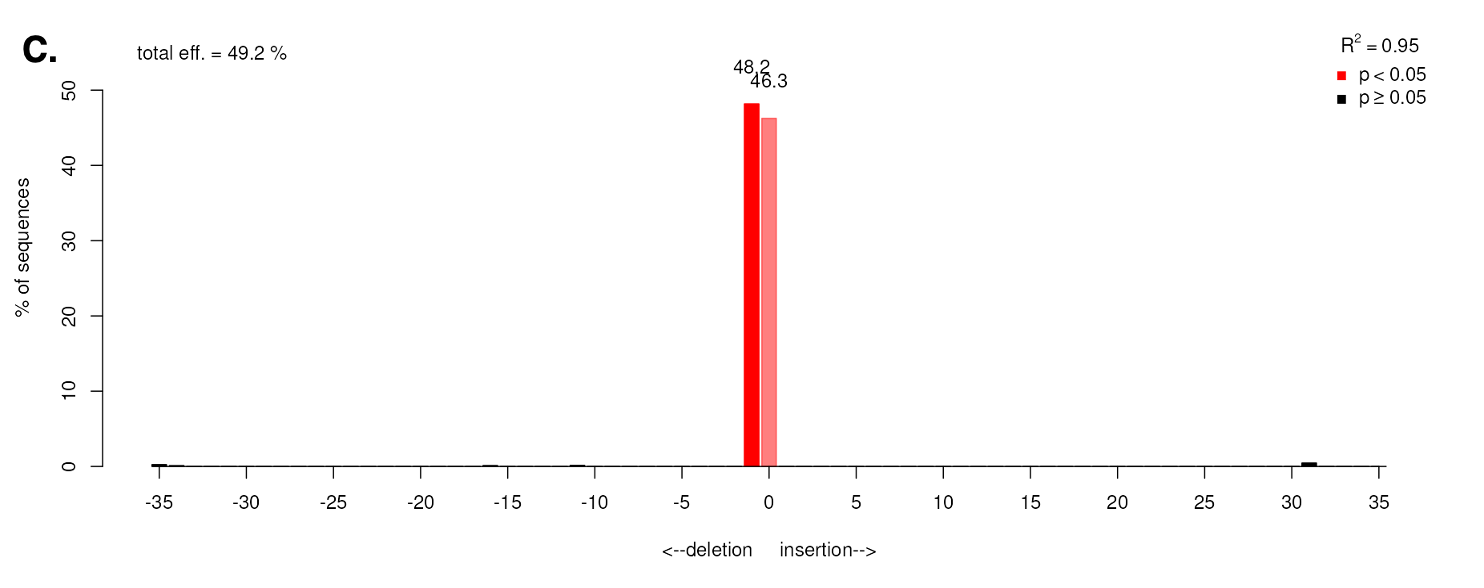
**

**A.** Simple sequence alignment showing the single base deletion in U2OS cell line D4 (*DDX52*^+/-^) compared with the sequence compared from the wild type U2OS cells.

**B and C.** Decomposition and TIDE DNA sequence analysis of the cell line D4 sequence, compared with the wild type cells, confirming that position nucleotide 282 has undergone the mutation.

**Supplementary Tables**

**Table S1: Plasmids used in this work.**

| Name | Description |
| --- | --- |
| pAP1 | pETDuet-1 with incorporated DDX52 ORF (Gene optimised) |
| pAP5 | pAP1 with DDX52 motif ll sequence mutated to encode D318A, D321A |
| pAP17 | pAP1 with DDX52 ORF mutated to encode F28_T128del |
| pPS2 | N-terminus DDX52 truncation in pETDuet |
| pPS3 | C-Terminus DDX52 truncation in pETDuet |
| pEGFP | GFP plasmid optimised for expression in human cells. |

**Table S2: Primers used to generate mutants.**

| Name | Mutant | 5’ to 3’ Sequence |
| --- | --- | --- |
| AP27 | DDX52^D318A+D321A^ | agcgcaAAATTGTTCGAAGACGGTAAAAC |
| AP28 |  | ttctgcCACCACAAGCCACTCCAC |
| AP94 | DDX52^p.V483_S599del^ | AACAGGATCCACGGTCGAACAGGA |
| AP95 |  | AGCAGGAAGCTTTTACGACTTATCCTC |
| AP96 | DDX52^p.F28_V128del^ | AGTGGTAAATTGGAGAATCTTCGCAAG |
| AP97 |  | AAAGCGCGCGGCATC |
| AP98 | DDX52^p.M1_V383del^ | AAGCTTGCGGCCGCATAATGC |
| AP99 |  | TTAAGCGCTATTACGCGCCCC |

**Table S3: Oligonucleotides used in assays.**

| Assay | Name | 5’ to 3’ Sequence |
| --- | --- | --- |
| Helicase | MW12 | TCGGATCCTCTAGACAGCTCCATGATCACTGGCACTGGTAGAATTCGGC |
|  | MW14 | [Cy5]CAACGTCATAGACGATTACATTGCTACATGGAGCTGTCTAGAGGATCCGA |
|  | PM16 | TGCCGAATTCTACCAGTGCCAGTGAT |
|  | PM17 | TAGCAATGTAATCGTCTATGACGTTG |
|  | MW12-FRET | [Cy5]TCGGATCCTCTAGACAGCTCCATGATCACTGGCACTGGTAGAATTCGGC |
|  | MW14-FRET | CAACGTCATAGACGATTACATTGCTACATGGAGCTGTCTAGAGGATCCGA[Cy3] |
| D and R-loop assays | PM4 | GGGTGAACCTGCAGGTGGGCGGCTGCTCATCGTAGGTTAGTTGGTAGAATTCGGCAGCGTC |
|  | RGL19 | GACGCTGCCGAATTCTACCAGTGCCTTGCTAGGACATCTTTGCCCACCTGCAGGTTCACCC |
|  | PM6-DNA | [Cy5]AAAGATGTCCTAGCAAGGCAC |
|  | PM6-RNA | [Cy5]AAAGAUGUCCUAGCAAGGCAC |
|  | PM7-DNA | [Cy5]AAAGATGTCCTAGCAAGGCACGATCGAGCGGATATCTATGACCAT |
|  | PM7-RNA | [Cy5]AAAGAUGUCCUAGCAAGGCACGAUCGAGCGGAUAUCUAUGACCAU |
| Annealing | ELB 40 | GGAGCTCCCTAGGCAGGATCGTTCGCGACGATGGCCTTCGAAGAGCTCCAGTTACGGATACGGATCCTGC |
|  | ELB 41 | [Cy5]GCAGGATCCGTATCCGTAACTGGAGCTCTTCGAAGGCCATCGTCGCGAACGATCCTGCCTAGGGAGCTCC |
|  | ELB 40 RNA | GGAGCUCCCUAGGCAGGAUCGUUCGCGACGAUGGCCUUCGAAGAGCUCCAGUUACGGAUACGGAUCCUGC |
|  | ELB40-FRET | GGAGCTCCCTAGGCAGGATCGTTCGCGACGATGGCCTTCGAAGAGCTCCAGTTACGGATACGGATCCTGC[Cy3] |
| EMSA | AP33 | [Cy5]UCAUAGACGAUUACAUUGCUACAUGGAGCUGUCUAGAGGAUCCGA |
|  | AP122 | [Cy5]TCATAGACGATTACATTGCTACATGGAGCTGTCTAGAGGATCCGA |
| Fluorescent anisotropy | Poly(T)35 (For FP assay) | [FAM]TTTTTTTTTTTTTTTTTTTTTTTTTTTTTTTTTTT |
|  | AP137 (Poly U35 for FP assay) | [FAM]UUUUUUUUUUUUUUUUUUUUUUUUUUUUUUUUUUU |

**Table S4: Bacterial strains.**

| Name | Genotype |
| --- | --- |
| Dh5𝛂 | fhuA2Δ(argF-lacZ)U169 phoA glnV44 Φ80Δ(lacZ)M15 gyrA96 recA1 relA1 endA1 thi-1 hsdR17 |
| BL21-AI | F^–^*omp*T *hsd*S_B_ (r_B_^–^, m_B_^–^) *gal dcm ara*B::T7RNAP-*tet*A |

**Table S5: gRNAs used in generation of CRISPR mutants.**

| Target | 5’ to 3’ Sequence |
| --- | --- |
| DDX52 Exon 4 | GCTTGCATTTGGATTGGCGT |
| DDX52 Exon 5A | TGGCTGGCAAGTTCTCGTGT |
| DDX52 Exon 5B | CTCGTGTTGGTGATATAATC |

**Table S6: Primers used for amplification of exons in CRISPR editing**

| Name | Target | 5’ to 3’ Sequence |
| --- | --- | --- |
| AP51 | DDX52 Exon 4 Forward | TGGAAGAACTGCAGTGTGGG |
| AP52 | DDX52 Exon 4 Reverse | ATGGGTTCAATGCCTGCCTT |
| AP53 | DDX52 Exon 5 Forward | TGTCCAAGCAGGGCTATATT |
| AP54 | DDX52 Exon 5 Reverse | ACCTTCCCTAGTGATTGAACA |
| AP66 | Off target site 1 forward | TCGTCGGCAGCGTCGCAGCAGCCTGGTTCTCGTGG |
| AP67 | Off target site 1 reverse | GTCTCGTGGGCTCGGGCCGATGGCCTTCCCCACAC |
| AP68 | Off target site 2 forward | TCGTCGGCAGCGTCTCCAATATCCGAGAAAGGAAACCT |
| AP69 | Off target site 2 reverse | GTCTCGTGGGCTCGGTGCAACTGAACACAATGGAAGTCA |
| AP70 | Off target site 3 forward | TCGTCGGCAGCGTCAGGCCACATACCACCCAGCATCA |
| AP71 | Off target site 3 reverse | GTCTCGTGGGCTCGGACCATGCAGCCTCAGTAGCTGCC |

**DDX52 Optimised ORF used for expression of human DDX52 and for site-directed mutagenesis. The sequence excludes the initial Methionine and the N-terminal (Histidine)_6_-tag**

GACGTACATGACCTGTTTCGCCGTCTTGGAGCCGGGGCAAAGTTTGATACACGCCGTTTTTCGGCGGATGCCGCGCGCTTTCAAATCGGCAAACGTAAGTATGATTTCGATAGTTCTGAGGTCTTACAGGGATTGGATTTCTTTGGAAATAAAAAGTCTGTACCAGGTGTTTGTGGAGCTTCCCAGACCCACCAGAAGCCGCAGAACGGGGAGAAAAAGGAAGAGTCCCTGACAGAGCGCAAGCGTGAACAATCCAAGAAGAAGCGTAAAACGATGACCAGCGAGATTGCCTCACAGGAGGAAGGTGCGACAATTCAGTGGATGTCATCTGTTGAGGCGAAAATCGAGGATAAAAAGGTCCAACGCGAAAGCAAACTGACCAGTGGTAAATTGGAGAATCTTCGCAAGGAGAAAATTAATTTCTTACGTAATAAGCACAAGATCCATGTACAAGGGACTGACCTGCCGGACCCCATTGCGACATTTCAGCAGTTAGACCAAGAATATAAAATTAACTCCCGTTTGTTACAGAATATTCTTGACGCCGGATTTCAGATGCCGACACCCATTCAAATGCAAGCCATCCCAGTGATGTTGCATGGACGCGAACTTCTTGCATCCGCACCGACAGGATCGGGAAAGACTTTAGCTTTCTCTATCCCAATTTTAATGCAATTAAAGCAACCCGCGAACAAAGGGTTTCGCGCGCTGATCATCTCTCCCACGCGTGAGTTGGCCAGCCAAATCCACCGTGAACTTATTAAGATCAGCGAAGGCACGGGGTTTCGTATTCATATGATTCATAAGGCAGCCGTGGCCGCTAAGAAATTTGGTCCAAAATCGTCTAAAAAATTTGATATTCTTGTCACGACCCCCAATCGCCTTATCTACTTGTTGAAGCAAGACCCGCCCGGCATCGACCTTGCCTCGGTGGAGTGGCTTGTGGTGGATGAATCCGATAAATTGTTCGAAGACGGTAAAACGGGCTTTCGTGACCAATTGGCCAGTATCTTTCTGGCCTGTACGAGTCATAAAGTTCGTCGTGCGATGTTCTCGGCAACGTTCGCCTACGACGTTGAGCAGTGGTGTAAATTGAACCTTGACAATGTTATTTCCGTGTCAATTGGGGCGCGTAATAGCGCTGTCGAAACGGTCGAACAGGAACTGCTTTTCGTCGGATCAGAAACGGGAAAATTGTTAGCCATGCGCGAGTTAGTCAAAAAAGGGTTCAACCCGCCGGTTCTTGTCTTTGTCCAGTCGATCGAACGTGCAAAGGAGCTGTTCCATGAATTAATCTACGAGGGAATTAATGTCGACGTAATTCATGCAGAACGTACTCAACAGCAGCGCGATAATACAGTTCATAGCTTTCGCGCTGGAAAAATCTGGGTCTTAATTTGCACGGCACTTCTGGCCCGTGGAATTGACTTCAAAGGGGTAAACCTTGTGATCAATTATGACTTTCCGACTTCTAGCGTAGAATATATCCATCGTATCGGTCGCACCGGACGTGCCGGGAACAAAGGTAAGGCCATCACTTTTTTTACTGAAGACGACAAGCCACTTTTACGCAGCGTGGCAAACGTGATCCAGCAAGCAGGATGTCCGGTGCCAGAGTACATTAAAGGCTTTCAGAAATTGTTGAGCAAGCAGAAAAAGAAGATGATTAAGAAGCCTCTGGAACGCGAATCTATTTCAACGACACCTAAATGCTTCTTAGAAAAGGCCAAGGACAAGCAGAAGAAGGTGACCGGCCAGAATAGTAAAAAAAAAGTCGCGTTAGAGGATAAGTCGTAA
